# Familial Adenomatous Polyposis and Desmoid Tumor Treated with Multivisceral Transplantation and Kidney Autotransplantation: Case Report and Literature Review

**DOI:** 10.1155/2019/6064720

**Published:** 2019-12-22

**Authors:** Libor Janousek, Robert Novotny, Michal Kudla, Martin Oliverius, Petr Wohl, Joan Minguet, Jan Martinek, Tomas Hucl, Jiri Fronek

**Affiliations:** ^1^Transplant Surgery Department, Institute for Clinical and Experimental Medicine, Prague, Czech Republic; ^2^Department of Diabetology, Institute for Clinical and Experimental Medicine, Prague, Czech Republic; ^3^Institute for Research and Medicine Advancement, Barcelona, Spain; ^4^Department of Hepatology, Institute for Clinical and Experimental Medicine, Prague, Czech Republic; ^5^Department of Anatomy, Second Faculty of Medicine, Charles University in Prague, Czech Republic

## Abstract

**Introduction:**

Desmoid tumours (DT) are commonly associated with Gardener's syndrome. Their surgical resection may be complicated by their close proximity to major vessels, multiple organ involvement, and frequent local recurrence. Multivisceral transplantation (MVTx) is an alternative treatment for patients with intestinal and liver failure. In patients with DT closely associated with renal structures but without end-stage kidney disease, concomitant excision of the patient's own kidney, ex vivo tumour resection with nephron-sparing surgery, or autotransplantation has been proposed.

**Case Presentation:**

A 36-year-old Caucasian female weighing 60 kg with Gardener's syndrome with a history of abdominal surgery was presented to our department with progressive abdominal distention associated with paroxysmal pain. With the use of CT, the patient was diagnosed with a mass arising from the mesenterial region. The patient had normal kidney function and nonalcoholic steatohepatitis. The patient was indicated for MVTx.

**Management and Outcome:**

After 16 months on the waiting list, the patient received a multivisceral graft from a deceased donor. Following the restoration of graft vascular flow, the patient's right kidney was removed and the DT dissected ex vivo before autotransplantation into the right pelvic fossa. The patient received immunosuppressive, antithrombotic, and antibiotic treatment. There was no acute rejection, though the patient experienced pulmonary infection, dysphagia, and oesophageal reflux with fungal infection. The patient had required temporary dialysis for acute renal failure for 75 days. One year after the surgery, nausea and violent vomiting caused delayed gastric emptying caused by spastic pylorus. Clinical improvement was achieved using gastric peroral endoscopic myotomy (G-POEM).

**Conclusion:**

MVTx with kidney autotransplantation is a feasible treatment option in patients with familiar adenomatous polyposis complicated by an abdominal DT. Precise tumour dissection with nephron-sparing surgery was carried ex vivo. G-POEM was used to relieve MVTx-related gastroparesis. The patient had no disease reoccurrence after one-year follow-up.

## 1. Introduction

The risk of desmoid tumour (DT) occurrence in patients with familial adenomatous polyposis (FAP) has been estimated to be 852 times that of the general population [1]. Approximately 12% of FAP patients develop these palpable solid masses. They occur most commonly in the intra-abdominal cavity, abdominal wall, or mesenteric region [2]. DT are benign, though locally invasive, unpredictable, and sometimes life threatening [3, 4]. Disease progression may result in complications such as chronic abdominal discomfort and a loss of nutritional autonomy leading to a dependency on lifelong total parenteral nutrition (TPN) [4]. Surgical excision is the most common treatment for DT that are symptomatic or close to vital structures. However, this is complicated by the proximity of tumours to major vessels, multiple organ involvement, and a high rate of local recurrence [5–7]. Consequently, multivisceral transplantation (MVTx) may be considered a more effective curative approach, especially given the extensive bowel pathology, multiple prior resections, and liver steatosis (as a result of TPN) commonly seen in end-stage FAP patients [8].

## 2. Case Presentation

The patient was a 36-year-old Caucasian female weighing 60 kg with Gardener's syndrome FAP subtype with progressive abdominal distention associated with paroxysmal pain. The patient had a history of abdominal surgery: right colon resection, right ureter resection, and multiple elective exploratory laparotomies for abdominal revision, including DT resections.

**Figure 1:**
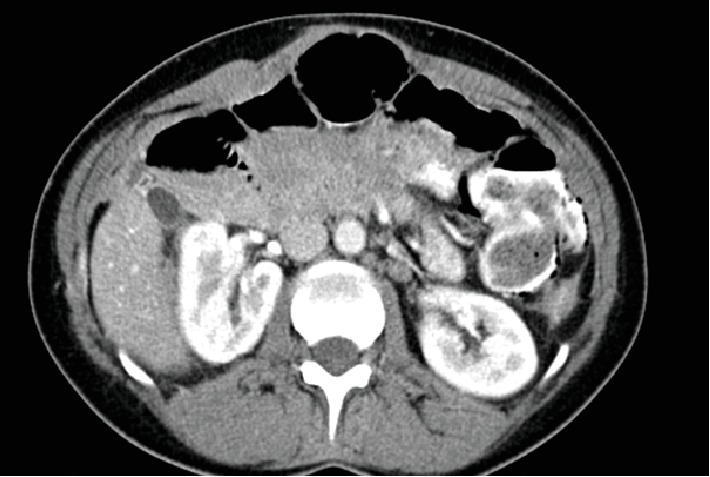
Intra-abdominal desmoid tumour.

Upon admitting, the patient underwent an abdominal-pelvic computed tomography examination that revealed an intra-abdominal mass 10 × 14 cm in size. The intra-abdominal mass embedded the uterus, the internal and external iliac vessels, and the coeliac and mesenteric arteries. The patient was diagnosed with mesenteric fibromatosis (Figure 1). Furthermore, kidney function tests (creatinine: 88 *μ*mol/l; urea: 5.5 mmol/l) were normal, rendering the patient ineligible for kidney transplantation. The scores for liver function according to the Nonalcoholic Steatohepatitis (NASH) Clinical Research Network histological scoring system for nonalcoholic fatty liver disease (NAFLD) are as follows: steatosis—2; lobular inflammation—1; hepatocellular ballooning—1; and fibrosis stage—0 to 1C [9]. The patient was indicated for MVTx (with liver) and placed on a waiting list.

### 2.1. Donor and Graft Preparation

The graft donor was a 63 kg, 18-year-old Caucasian female. The cause of death was acute intracerebral haemorrhage as a result of aneurysm rupture. The donor died in a hospital. Both the donor and recipient were blood group ABO: A and HLA: negative. T-cell-cross-match and B-cell-cross-match were both negative.

A multivisceral graft consisting of the stomach, duodenum, pancreas, liver, small bowel, and spleen was then harvested “en bloc.” All organs were flushed, topically cooled on ice, and perfused with ViaSpan® (Belzer UW) cold storage transplant solution. During back-table preparation, vascular cuffs for inflow (aortal conduit) and outflow (vena cava inferior) were established on the graft. The total cold ischemic time was 17 minutes, with a warm ischemic time of 1 minute. Donor basic characteristics are summarised in Table 1.

### 2.2. Recipient Surgery

To achieve optimal abdominal exposure, the patient was placed in a supine position. A midline laparotomy was used for surgical access. A proximal gastrointestinal transection was carried out at the level of the distal oesophagus, before proceeding with total abdominal multivisceral resection “en bloc.” This included the removal of the whole stomach, liver, pancreaticoduodenal complex, spleen, small intestine remnant, and right hemicolon. The donor graft was placed in an orthotopic position and vascular flow secured via end-to-side anastomosis of the graft infrarenal aorta to that of the recipient (inflow) and side-to-side anastomosis of the graft retrohepatic vena cava inferior to that of the recipient (outflow). Retroperitoneal lymph node dissection was then performed.

The right kidney was removed and perfused with ViaSpan® cold storage solution. The DT was dissected away from the ureter and hilum ex vivo. The kidney was then autotransplanted into the patient's right pelvic fossa. The total ischemic time for the kidney was 17 minutes, with warm ischemic time accounting for 1 minute.

Lastly, restoration of intestinal continuity was achieved via esophagogastrostomy, pyloroplasty, and terminal ileostomy. A gastrostomy tube and enteral feeding tube were placed at the end of the procedure. In this instance, a skin-only closure in the upper quarter was performed, resulting in an abdominal hernia. The entire surgical procedure took 9 hours. The patient was transferred to the intensive care unit (ICU) to recover. Recipient basic characteristics are summarised in Table 1.

### 2.3. Postoperative Course

The immunosuppression protocol was started one day after the surgery and consisted of antilymphocyte induction with alemtuzumab 60 mg/5 days (Genzyrne Corp., Cambridge, MA) combined with tacrolimus 16 mg/day and prednisone 10 mg daily dose. The patient was taking the following prophylactic antibiotics: valganciclovir 450 mg daily dose and sulfamethoxazole-trimethoprim 480 mg daily dose. Aspirin 100 mg daily dose was prescribed to prevent thrombosis.

The postoperative course was complicated by several events: pulmonary infection and respiratory insufficiency due to pleural effusions, hyperhydration, and acute renal failure requiring temporary dialysis (75 days). On the 18th postoperative day, the patient underwent exploratory laparotomy due to an intra-abdominal infected haematoma and the migration of the gastrostomy tube from its original position. The haematoma was evacuated through an opening in the midline laparotomy. She also suffered from dysphagia and gastroesophageal reflux, though no acute rejection was seen. The patient was discharged from the hospital in the third postoperative month.

Within the first year after transplantation, the patient required readmission on multiple occasions for oesophageal reflux with a fungal infection, dehydration, and ureteral stent extraction. One year after the MVTx, the patient became nauseous and experienced violent vomiting. Upper endoscopy failed to identify any evidence of ulcerative lesions and gastric outlet obstruction. Scintigraphy identified delayed gastric emptying and spastic pylorus. Therefore, a gastric peroral endoscopic myotomy (G-POEM) was performed. Clinical improvement was observed by scintigraphy five days later. The patient was discharged on the 7th postadmission day. The following week, the patient was readmitted with anaemia (Hb: 69 g/L. Htc: 0,206). Endoscopy revealed ulceration and dehiscence of the layers of the G-POEM incision. This was identified as the source of bleeding. The ulcer was treated with conservative therapy and monitored through regular endoscopy, with a resolution by day 14. No evidence of recurrent disease was seen via a CT scan at 1-year follow-up.

## 3. Discussion

DT arise from fascial or deep musculoaponeurotic structures. They can be classified as extra-abdominal, abdominal wall, and intra-abdominal types [10]. The etiology of DT is still unclear. Approximately 2% of DT are associated with FAP caused by mutation in the FAP gene that is located in chromosome 5q21, or Gardner's syndrome [11, 12]. Known risk factors associated with DT are as follows: prior abdominal surgery, typically prophylactic colectomy, localization of the FAP germline mutation, female sex, and a family history of DT [13]. DT account for 10-14% of deaths in FAP patients [14].

DT are usually presented as a painless or minimally painful mass with a history of slow growth [10]. Intra-abdominal DT are large neoplasms that remain asymptomatic until their size causes visceral compression. Symptoms of intestinal, ureteric, vascular, or neural obstruction are often the initial clinical manifestations [4, 10]. DT do not metastasize. Therefore, morbidity and mortality of the intra-abdominal DT depend primarily on their localization [15].

To date, there are no clear guidelines on the treatment of intra-abdominal DT. In patients with stable or spontaneously regressing DT, the wait-and-see policy is a widely accepted strategy. However, patients with progressive or symptomatic DT should undergo a surgical intervention [16].

The first G-POEM, performed for the treatment of refractory gastroparesis, was performed by Gonzales et al. in 2015 [17]. This novel and minimally invasive approach has been recently shown to be effective in a small multicentre study of 30 patients with diabetic, postsurgical, or idiopathic gastroparesis, with 86% of patients achieving normal gastric emptying by 5.5 months [18].

Initially, the success of visceral transplantation was severely hampered by allograft rejection due to high lymphoid tissue content and digestive system complexity. Nowadays, safety has been greatly improved by advancements in immunosuppressive medicine, surgical technique, and postoperative management [19–21]. Visceral transplantation is considered the standard of care for patients with combined complex abdominal pathology and nutritional failure, divisible into four subtypes: intestine only; intestine, liver, and pancreas; MVTx (en bloc intestine, pancreas, liver, stomach, and duodenum with/without kidney and/or colon); and modified MVTx (liver exclusion) [21].

Several other small studies have also shown patients with slow-growing intra-abdominal malignancies to be rescuable with MVTx, with more recent studies reporting less frequent mortality [8, 11, 21]. In patients with fully functioning kidneys, the close proximity of a DT to key renal structures may preclude its safe removal in situ. In this complicated situation, donor MVTx (without the kidney) followed by excision of the patient's own kidney, ex vivo tumour resection, and nephron-sparing surgery, and autotransplantation is an option. Workbench dissection allows a greater likelihood of obtaining a tumour-free resection margin and preserving renal function [22]. The use of renal autotransplantation has been reported for a range of conditions, including urologic tumours [23]. Kato et al. reported a MVTx in a patient with Gardener's syndrome experiencing recurring DT in his skin, the muscle layer, and intra-abdominal cavity, but not in donor tissue [24].

To the best of our knowledge, this is the first time autotransplantation of the patient's kidney has also been performed with MVTx for DT. Carrying out the resection of the DT at the workbench was helpful for two reasons. Firstly, it allowed negative tumour margins to be obtained more easily. This is likely to have contributed to the lack of recurrence observed at one year, given that the quality of surgical margins has been identified as a key factor determining postoperative recurrence rate in patients with DT [7]. Secondly, the extracorporeal setting permitted nephron-sparing surgery to be performed with precision, which would otherwise have been challenging or impossible in situ. The lack of permanent renal failure experienced by our patient postprocedurally is likely linked to this strategy. Reducing the need for long-term haemodialysis is particularly important, as it has a direct impact on a patient's quality of life and mortality risk [25].

In the present case report, we describe the case of a FAP patient with DT and preserved renal function, treated with MVTx with kidney autotransplantation. The present case adds to the pool of evidence for the feasibility of MVTx as a life-saving procedure, despite the extensive dissection and operation time. Despite the state of liver fibrosis, we had decided to go for MVTx including a liver graft as liver fibrosis is a progressive disease and the patient had already been through a couple of abdominal surgeries; thus, liver transplantation after modified MVTx would be surgically extremely difficult with a very high risk of perioperative complications [21].

## 4. Conclusion

MVTx with kidney autotransplantation is a feasible treatment modality for a complicated abdominal DT in a patient with Gardener's syndrome resulting in a lack of graft rejection and survival without disease recurrence at one year. Precise tumour dissection away from the ureter and nephron-sparing surgery were performed ex vivo, likely contributing to the patient's long-term preservation of renal function. Thus, MVTx plus kidney autotransplantation may be an attractive procedure for patients indicated for MVTx who do not fulfil renal failure criteria but who have DT closely associated with kidney structures. Finally, G-POEM was used safely and effectively to relieve MVTx-related gastroparesis and may be considered for future intestinal transplant patients with dysphagia and delayed gastric emptying.

## Figures and Tables

**Table 1:** Recipient and deceased donor characteristics.

	Recipient	Deceased donor
Age (years)	36	18
Weight (kg)	60	63
Height (cm)	162	165
Blood type	A-	A-
HLA phenotype	AA1, 2/B8, 38/Cw7/DR3, 13	A24, 31/B49/DR15, 16
Total ischemic time—kidney (min)	17	NA

## References

[1] Righetti A. E., Jacomini C., Parra R. S., de Almeida A. L., Rocha J. J., Féres O. (2011). Familial adenomatous polyposis and desmoid tumors. *Clinics (São Paulo, Brazil)*.

[2] Bazemore T. C., Maskarinec S. A., Zietlow K., Hendershot E. F., Perfect J. R. (2016). Familial adenomatous polyposis manifesting as *Lactococcus* endocarditis: a case report and review of the association of *Lactococcus* with underlying gastrointestinal disease. *Case Reports in Infectious Diseases*.

[3] Ono H., Hori K., Tashima L., Tsuruta T., Nakatsuka S. I., Ito K. (2018). A case of retroperitoneal desmoid-type fibromatosis that involved the unilateral ureter after gynaecologic surgery. *International Journal of Surgery Case Reports*.

[4] Quintini C., Ward G., Shatnawei A. (2012). Mortality of intra-abdominal desmoid tumors in patients with familial adenomatous Polyposis. *Annals of Surgery*.

[5] Jenayah A. A., Bettaieb H., Saoudi S. (2015). Desmoid tumors: clinical features and treatment options: a case report and a review of literature. *The Pan African Medical Journal*.

[6] Bonvalot S., Desai A., Coppola S. (2012). The treatment of desmoid tumors: a stepwise clinical approach. *Annals of Oncology*.

[7] Wang Y.-f., Guo W., Sun K.-k. (2015). Postoperative recurrence of desmoid tumors: clinical and pathological perspectives. *World Journal of Surgical Oncology*.

[8] Nikeghbalian S., Aliakbarian M., Shamsaeefar A., Kazemi K., Bahreini A., Malekhosseini S. A. (2013). Multivisceral transplantation for the treatment of intra-abdominal tumors. *Transplantation Proceedings*.

[9] Kleiner D. E., Brunt E. M., van Natta M. (2005). Design and validation of a histological scoring system for nonalcoholic fatty liver disease. *Hepatology*.

[10] Mastoraki A., Schizas D., Vergadis C. (2019). Recurrent aggressive mesenteric desmoid tumor successfully treated with sorafenib: a case report and literature review. *World Journal of Clinical Oncology*.

[11] Mullen J. T., DeLaney T. F., Rosenberg A. E. (2013). *β*-Catenin mutation status and outcomes in sporadic desmoid tumors. *The Oncologist*.

[12] Leal R. F., Silva P. V., Ayrizono Mde L., Fagundes J. J., Amstalden E. M., Coy C. S. (2010). Desmoid tumor in patients with familial adenomatous polyposis. *Arquivos de Gastroenterologia*.

[13] Desurmont T., Lefèvre J. H., Shields C., Colas C., Tiret E., Parc Y. (2015). Desmoid tumour in familial adenomatous polyposis patients: responses to treatments. *Familial Cancer*.

[14] de Camargo V. P., Keohan M. L., D'Adamo D. R. (2010). Clinical outcomes of systemic therapy for patients with deep fibromatosis (desmoid tumor). *Cancer*.

[15] Chen C. B., Chiou Y. Y., Chen C. H., Chou Y. H., Chiang J. H., Chang C. Y. (2010). Sonographic and computed tomography findings of intra-abdominal desmoid tumor. *Journal of the Chinese Medical Association*.

[16] Smith A. J., Lewis J. J., Merchant N. B., Leung D. H., Woodruff J. M., Brennan M. F. (2000). Surgical management of intra-abdominal desmoid tumours. *The British Journal of Surgery*.

[17] Gonzalez J. M., Vanbiervliet G., Vitton V. (2015). First European human gastric peroral endoscopic myotomy, for treatment of refractory gastroparesis. *Endoscopy*.

[18] Khashab M. A., Ngamruengphong S., Carr-Locke D. (2017). Gastric per-oral endoscopic myotomy for refractory gastroparesis: results from the first multicenter study on endoscopic pyloromyotomy (with video). *Gastrointestinal Endoscopy*.

[19] Peaudecerf L., Rocha B. (2011). Role of the gut as a primary lymphoid organ. *Immunology Letters*.

[20] Todo S., Tzakis A., Abu-Elmagd K. (1995). Abdominal multivisceral transplantation. *Transplantation*.

[21] Bharadwaj S., Tandon P., Gohel T. D. (2017). Current status of intestinal and multivisceral transplantation. *Gastroenterology Report*.

[22] Nikeghbalian S., Aliakbarian M., Kazemi K. (2014). Ex-vivo resection and small-bowel auto-transplantation for the treatment of tumors at the root of the mesentery. *International journal of organ transplantation medicine*.

[23] Azhar B., Patel S., Chadha P., Hakim N. (2015). Indications for renal autotransplant: an overview. *Experimental and Clinical Transplantation*.

[24] Kato T., Ruiz P., Thompson J. F. (2002). Intestinal and multivisceral transplantation. *World Journal of Surgery*.

[25] Kemmer H., Siemer S., Stockle M. (2007). Nephrectomy, Work Bench Surgery, and Autotransplantation: A Case of a Solitary Left Kidney with an Extensive Centrally Located Renal Cell Carcinoma and a Tumour Thrombus Entering the Vena Cava!(https://ars.els- cdn.com/content/image/1-s2.0-S0302283807000267-eulogo1.jpg). *European Urology*.

